# Construction of a machine learning-based risk prediction model for depression in middle-aged and elderly hypertensive people in China: a longitudinal study

**DOI:** 10.3389/fpsyt.2024.1398596

**Published:** 2024-05-03

**Authors:** Fangzhu Ai, Enguang Li, Qiqi Ji, Huijun Zhang

**Affiliations:** Department of Nursing, Jinzhou Medical University, Jinzhou, China

**Keywords:** hypertension, depression, middle-aged and elderly, machine learning, prediction model

## Abstract

**Background:**

Hypertension is a common chronic disease that can trigger symptoms such as anxiety and depression. Therefore, it is essential to predict their risk of depression. The aim of this study is to find the best prediction model and provide effective intervention strategies for health professionals.

**Methods:**

The study subjects were 2733 middle-aged and older adults who participated in the China Health and Retirement Longitudinal Study (CHARLS) between 2018 and 2020. R software was used for Lasso regression analysis to screen the best predictor variables, and logistic regression, random forest and XGBoost models were constructed. Finally, the prediction efficiency of the three models was compared.

**Results:**

In this study, 18 variables were included, and LASSO regression screened out 10 variables that were important for the establishment of the model. Among the three models, Logistic Regression model showed the best performance in various evaluation indicators.

**Conclusion:**

The prediction model based on machine learning can accurately assess the likelihood of depression in middle-aged and elderly patients with hypertension in the next three years. And by combining Logistic regression and nomograms, we were able to provide a clear interpretation of personalized risk predictions.

## Introduction

The aging of the population is one of the major social problems in the world today (1). As the country with the largest elderly population in the world, China is faced with severe challenges and threats of population aging (2). One of the biggest challenges of aging is health-related issues, most notably chronic disease (3). Admittedly, chronic disease has become one of the biggest threats to human health, especially to the elderly (4). Among these chronic diseases, cardiovascular diseases are the most common and the leading cause of death in China. High blood pressure is widely recognized as a major risk factor for cardiovascular disease and has one of the highest disease burdens globally (5). According to the World Health Organization (WHO), about 1.13 billion people worldwide currently have high blood pressure, and the number of people with high blood pressure is expected to increase by more than 400 million by 2025 (6). Over the past few decades, the prevalence of hypertension has increased dramatically, and disability-adjusted life years associated with hypertension have also increased significantly (7). In addition, the prevalence of hypertension among the elderly in China is as high as 50% (8). This situation has become one of the chronic diseases that seriously threaten public health, especially in the middle-aged and elderly population, and has brought a heavy burden to the whole society (9).

The increase in hypertension is accompanied by various problems and challenges. Studies have shown that individuals with high blood pressures have a higher prevalence of mental health disorders, due to changes in physical symptoms of high blood pressure patients, which seriously affect physical and mental health, prone to depression, anxiety and loneliness and other problems (10–12). In addition, taking medication every day and worrying about serious complications may also increase the psychological burden on patients (13). A meta-analysis study showed that the prevalence of depression in people with high blood pressure was 26.8%, higher than in the general population (14). Several studies in China have also suggested that high blood pressure is a risk factor for depression (15, 16). Studies have also shown that symptoms of depression are associated with higher rates of high blood pressure (17, 18). Many studies have shown that depression has a significant impact on hypertensive patients (19, 20). In addition, when the same scale was used to screen populations in different regions, the depression detection rate among hypertensive patients in China was higher than 17.3% in American Indians (21) and 24.0% in Australia (22).

Depression is a mood disorder that can cause a variety of functional physical impairments and loss of interest in daily activities, which can reduce quality of life (23). At present, depression has become a major mental health problem in low-income, middle-income, and high-income countries, and its lifetime risk has risen steadily over the past few decades (24, 25). According to Whiteford H. A. et al., they used data from the Global Burden of Diseases, Injuries, and Risk Factors Study 2010 to estimate the burden of disease associated with mental and substance use disorders (26). The findings show that mental and substance use disorders account for 7.4% of disability-adjusted life years globally. At the same time, mental and substance use disorders are also the main causes of global years of life lost due to disability, and depression contributes the most to the global years of life lost due to mental and substance use disorders, reaching 40.5%. The findings highlight the important impact of depression on global health. In addition, a 2013 systematic review of Gu L et al. showed that the lifetime prevalence of depression in China was 3.3% (27). In 2014, Smith K et al. showed the epidemiological status of depression in the world in Nature, among which the incidence of depression in China was 3.02% (28). Depression is expected to become the leading cause of the global burden of disease by 2030 and has become the leading cause of suicide in China. This series of data shows that depression has become a global public health problem that needs adequate attention and attention (28).

When older people suffer from both hypertension and depression, the double burden of physical and mental illness increases the risk of cardiovascular and cerebrovascular disease, non-compliance with medication, poor quality of life, and suicide (12, 29, 30). In addition, research has shown that depression can affect treatment, physical function, and health outcomes in people with high blood pressure (31). Patients with long-term hypertension often have emotional disorders, but few people can timely detect and correctly understand the severity of depression, thus delaying treatment, which will also affect the control of hypertension. There is a lot of research on high blood pressure and depression. In the early 1990s, some studies showed no clear correlation between high blood pressure and depression. However, recent studies have found that clinically significant depressive symptoms are independently associated with elevated blood pressure (32, 33). In addition, Chinese scholars have also studied the heterogeneity of depression in non-hospitalized elderly patients with hypertension and studied the influencing factors of various categories (34).

In summary, although there have been many studies on the association between high blood pressure and depression, there is still a lack of tools and practical applications for risk assessment in high-risk patients. Clinically, we can only rely on the summary of experience to promote the prevention of depression in hypertensive patients. However, with the continuous progress of artificial intelligence (AI) technology in recent years, it has become an important topic in the field of health care, and its application is also expanding. Machine learning (ML) is an in-depth study of how to extract valuable information from massive amounts of data. It is not only a theory but also a practical discipline, which is the core and one of AI (35, 36). ML was first proposed by Samuel in 1959 (37), through ML, we can extract the best objective function from a large amount of data and use these functions to achieve optimization. It can effectively help doctors predict diseases through ML, thus effectively supporting clinicians’ diagnosis and treatment decisions. Algorithms such as logistic regression (LR), random forest (RF), lasso regression, and XGBoost are included for the construction and validation of disease prediction models. However, in the prediction model of depression in middle-aged and elderly people with hypertension, there is a lack of relevant research. Therefore, this study will take the hypertension population in the China Health and Retirement Longitudinal Study database as the research object and use R studio software to organize and analyze the data. Three classical machine learning algorithms were used to scientifically construct a prediction model for depression risk in middle-aged and elderly hypertensive patients. By evaluating and screening the efficacy of the model, an optimal model algorithm is derived to better understand the patient’s disease changes.

## Materials and methods

### Data source

This study used data from the China Health and Retirement Longitudinal Study (CHARLS) from 2018 to 2020. CHARLS is a large-scale interdisciplinary survey project hosted by the National School of Development at Peking University. The project uses household surveys to collect high-quality longitudinal survey data from a nationally representative sample of people aged 45 years and older and their spouses (38, 39). The CHARLS survey covers 150 counties and 450 communities (villages) in 28 provinces. The survey used a probability sampling method proportional to population size, sampling at the county, household, and individual levels in four phases. This method has strong advantages in scientificity, geographical coverage, representativeness, and authenticity of samples.

### Study participants

**Figure 1:**
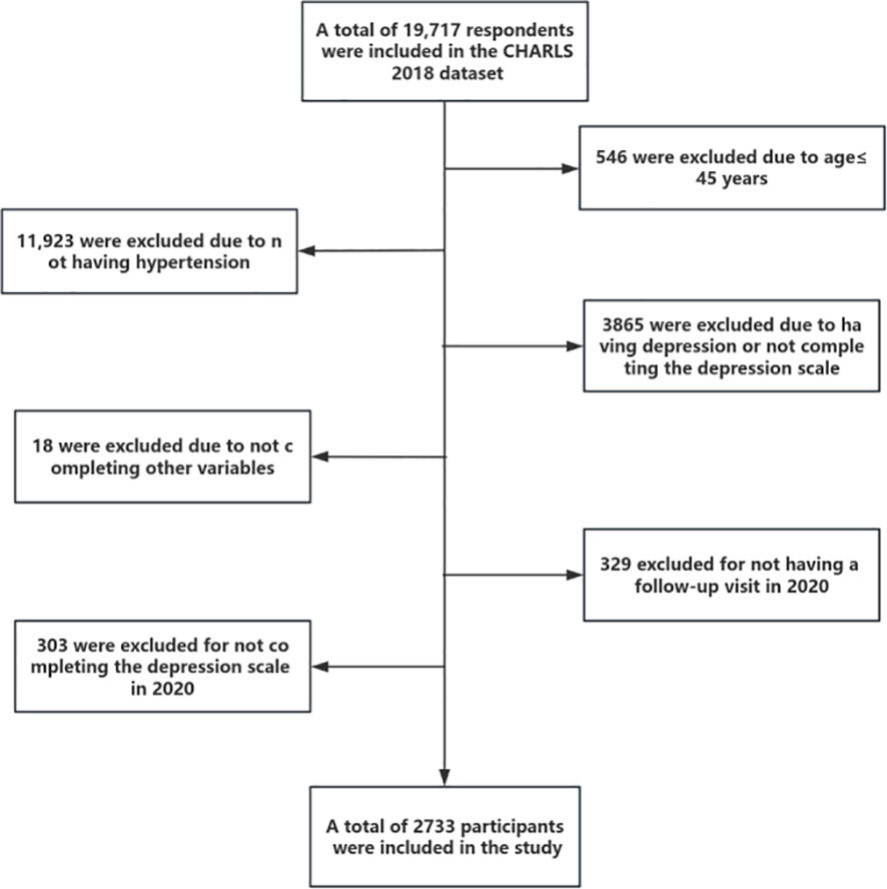
A flow chart for study population selection.

According to the purpose of the survey, the inclusion criteria for this study included: (1) age 45 years or older; (2) Patients with clinical diagnosis of hypertension. Exclusion criteria included: (1) participants who already had depressive symptoms or had not completed the depression scale at baseline (2018); (2) Participants who are unable to provide additional survey information on their own or who lack reliable information; (3) Participants who did not participate in follow-up in 2020; (4) Participants who did not complete the depression scale in 2020. In the end, a total of 2733 middle-aged and elderly people were included in this study, and the detailed process of participant selection is shown in **Figure 1**. The sampling unit for this study was middle-aged and elderly hypertensive patients. And since the number of samples screened in this study was large enough, no sampling weights were performed.

### Research variable

#### Outcome variable

In the CHARLS questionnaire, participants were screened for depressive symptoms using the CESD-10 scale. The scale has strong reliability and validity, especially in the measurement of depression symptoms in the elderly (40, 41). The scale consists of ten items and uses the Likert4 scoring method. Item 5 “I am hopeful for the future” and item 8 “I am happy” are positive items, and the rest are negative items. Negative entries are assigned according to “rarely or not at all (less than 1 day) =0”, “not much (1-2 days) =1”, “sometimes or half the time (3-4 days) =2” and “most of the time (5-7 days) =3”, while positive entries are assigned in reverse. Finally, the score of each item is added to get the total score, which ranges from 0 to 30 points. A total score of ≥10 was defined as the presence of depressive symptoms; If the total score is less than 10, it is defined as no depressive symptoms. A higher score indicates more severe depressive symptoms (42).

#### Socio-demographic factors

Sociodemographic factors include gender, hukou, education level, marital status, place of residence, and age. Among them, gender is divided into male and female; The level of education is classified as “none” and “ ≥1 year”; Types of hukou include “agricultural hukou” and “non-agricultural hukou”; Marital status can be classified as “married” (including living with a spouse or temporarily separated for reasons such as work) and “unmarried” (including separated, divorced or widowed); Place of residence are divided into “urban”, “urban-rural integration and special areas” and “rural”; Age is treated as a continuous variable.

#### Behavioral factors

Behavioral factors included exercise, social activities, smoke, drink, and sleep duration. Among them, exercise, social activities, smoke, and drink were classified as “yes” and “no”. Sleep duration was measured by asking the question, “During the past month, how many hours per night did you sleep on average?”. And was treated as a continuous variable in this study.

#### Health status

In this study, health status included disability, hearing difficulties, pain, and the number of chronic diseases. For the definition of disability, the following five questions were used: (1) Do you have one of the following disabilities? (2) Do you have Brain damage/intellectual disability? (3) Do you have vision problems? (4) Do you have hearing problems? (5) Do you have a Speech impediment? Participants were defined as disabled if they answered “yes” to at least one of the above questions, otherwise, it is “no”. For the definition of hearing difficulty, the following three questions were used: (1) Do you ever wear a hearing aid? (2)Is your hearing very good, good, fair, poor, or very poor(3)Do you have hearing problems? Hearing difficulties were defined if the answer to questions 1 or 3 was “yes” or if the answer to question 2 was “poor”. In this study, chronic diseases included 13 types, namely: dyslipidemia, diabetes, cancer, cardiovascular disease, chronic lung disease, liver disease, psychiatric problems, stroke, psychiatric illness, arthritis or rheumatic disease, kidney disease, digestive system disease, or asthma. The number of chronic diseases was treated as a continuous variable in this study. In addition, the pain was defined as “yes” and “no”.

#### Mental health factors

Mental health factors included life satisfaction, self-rated health, and self-rated memory, which were defined as “good”, “fair” and “bad” in this study, and all three variables are categorical in this study.

### Statistical analysis

In this study, R Studio software was used for data analysis with the aid of several R software packages, including rms, ROCR, Hmisc, random forest, glment, and caret, among others. Firstly, the general information table and scores of the patients were described. Because the measurement data in this study were not normally distributed, they were expressed as median and interquartile range, and the Mann-Whitney U test was used for comparison between groups and within groups. Count data were expressed as utilization rate, constituent ratio, or frequency, and the chi-square test was used for comparison between groups and within groups. The test level was set as α=0.05. Next, significant risk factors were screened using the Lasso regression method. Then, three machine learning algorithms, including logistic regression (LR), random forest (RF), and XGBoost, were used in the training set to construct depression risk prediction models for middle-aged and elderly hypertensive people.

Finally, the performance of the prediction models was evaluated by the area under the Receiver Operating Characteristic (ROC) curve, specificity, sensitivity, positive predictive value (PPV), and negative predictive value (NPV).

## Results

### Analysis of the number of patients

**Figure 2:**
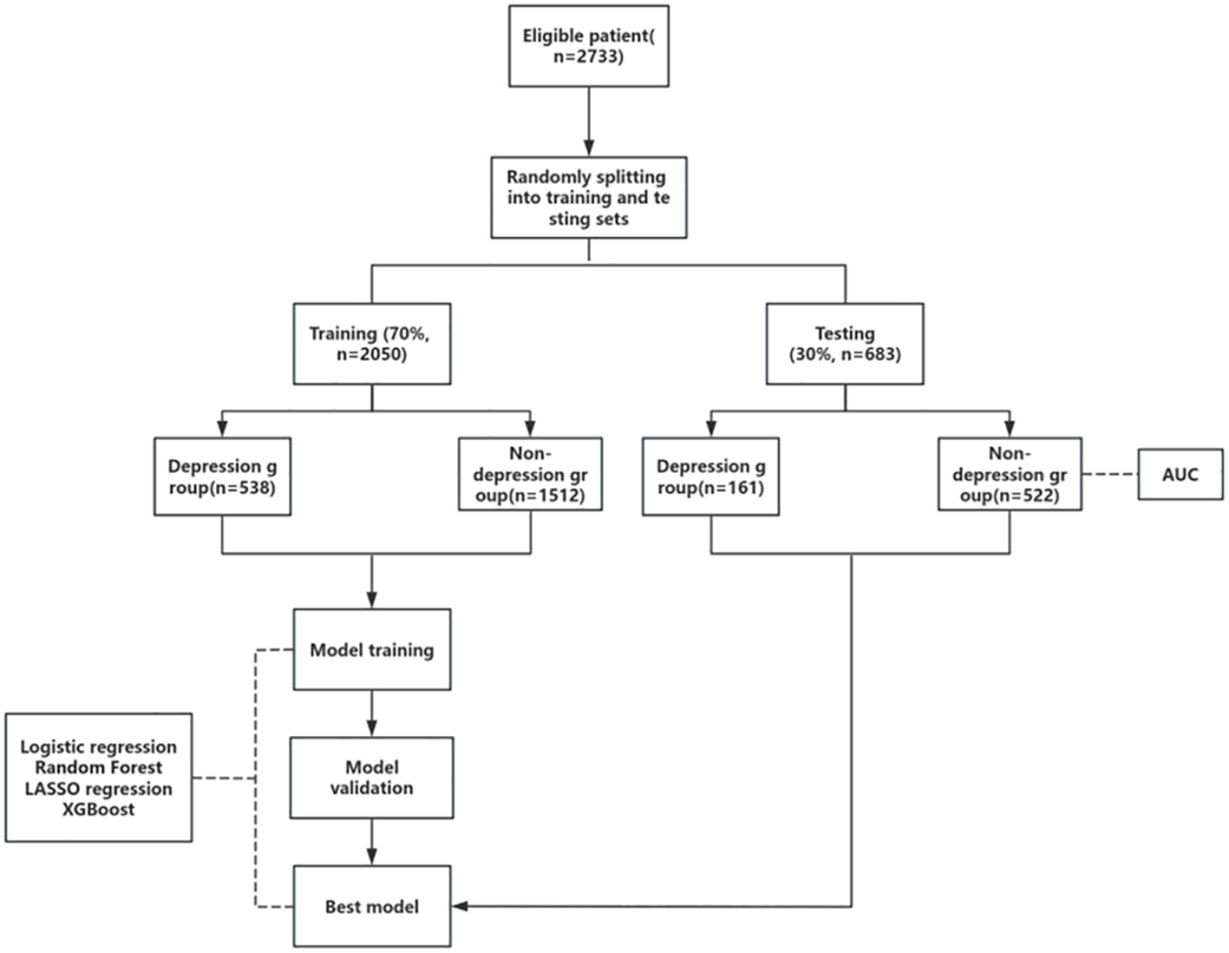
Flow chart of the study.

We randomly divided all the screened patients into a training set and a calibration set in a 7:3 ratio. The training set served as the modeling group with 2050 cases, and the calibration set served as the testing group with 683 cases. For the flow chart of the study, see **Figure 2**.

#### Baseline data of training set and testing set

Except for hearing difficulty, social activities and age, there were no significant differences in baseline characteristics between the two groups (P >0.05), indicating that the two groups would not be biased due to the uneven distribution of dependent variables, as detailed in **Table 1**.

**Table 1:** Comparison of baseline data between the two groups.

	Traning set	Testing set	H/χ2	*p*-value
**Gender**			0.136	0.712
Male	1151(56.1%)	389(57%)		
Female	899(43.9%)	294(43%)		
**Hukou**				0.402
Non-agricultural	1472(71.8%)	479(70.1%)	0.702	
Agricultural	578(28.2%)	204(29.9%)		
**Education level**				0.386
None	310(15.1%)	94(13.8%)		
≥1	1740(84.9%)	589(86.2%)		
**Marital Status**			0.019	0.890
No spouse	223(10.9%)	73(10.7%)		
Have a spouse	1827(89.1%)	610(89.3%)		
**Disabilities**			1.091	0.296
No	1406(68.6%)	483(70.3%)		
Yes	644(31.4%)	200(29.3%)		
**Hearing difficulty**			3.976	0.046
No	1797(87.7%)	618(90.5%)		
Yes	253(12.3%)	65(9.5%)		
**Pain**			1.359	0.244
No	899(43.9%)	317(46.4%)		
Yes	1151(56.1%)	366(53.6%)		
**Exercise**			0.457	0.499
No	129(6.3%)	48(7%)		
Yes	1921(93.7%)	635(93%)		
**Social activities**			3.921	0.048
No	1110(54.1%)	340(49.8%)		
Yes	940(45.9%)	343(50.2%)		
**Smoke**			0.225	0.614
No	1503(73.3%)	494(72.3%)		
Yes	547(26.7%)	189(27.7%)		
**Drink**			0.648	0.421
No	1234(60.2%)	423(61.9%)		
Yes	816(39.8%)	260(38.1%)		
**Resident**			1.918	0.383
Urban	493(24%)	179(26.2%)		
Rural-Urban Integration and Special Areas	189(9.2%)	68(10%)		
Rural	1368(66.7%)	436(63.8%)		
**Life satisfication**			4.735	0.094
Good	861(42%)	256(37.5%)		
Fair	1112(54.2%)	403(59%)		
Bad	77(3.8%)	24(3.5%)		
**Self reported memory**			1.631	0.442
Good	303(14.8%)	88(12.9%)		
Fair	1267(61.8%)	436(63.8%)		
Bad	480(23.4%)	159(23.3%)		
**Self reported health**			0.472	0.790
Good	461(22.5%)	156(22.8%)		
Fair	1181(57.6%)	384(56.2%)		
Bad	408(19.9%)	143(20.9%)		
**Sleep duration**	6.5(2.5)	6.5(3)	1361.52	0.832
**Number of chronic diseases**	2(2)	2(2)	1360.37	0.438
**Age**	63(13)	62(13)	1299.68	0.010

#### Identification of risk factors

**Figure 3:**
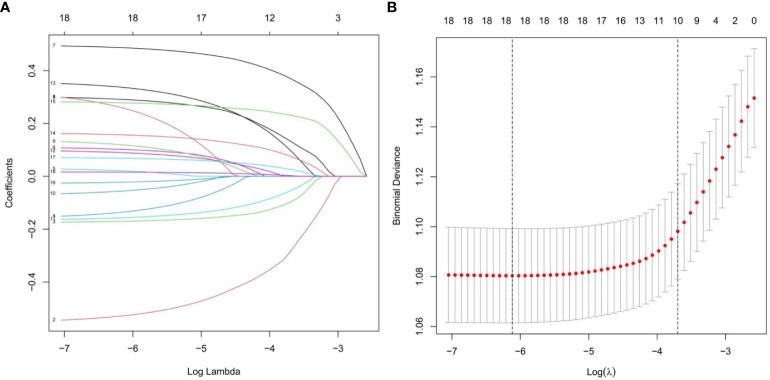
Lasso regression model was used to screen the risk factors. **(A)** Coefficient curves are generated according to the logarithmic (lambda) sequence, with nonzero coefficients generated by the optimal lambda. **(B)** Results of tenfold cross validation, partial likelihood deviation (binomial deviation) curves were plotted by validating the optimal lambda in the LASSO model, and virtual vertical lines were plotted according to the minimum lambda and standard error criteria.

In the training set, with the presence or absence of depression as the dependent variable (yes =1, no =0) and preselected risk factors for depression in middle-aged and elderly hypertensive patients as the independent variables, lasso Regression was used to screen risk factors. As can be seen from **Figure 3A**, the coefficients of the independent variables included in the model from the beginning will be gradually compressed, and the coefficients of the last part of the independent variables will be compressed to 0 to avoid overfitting the model. As can be seen from **Figure 3B**, by means of 10-fold cross-validation, λ+1 with the smallest error was selected as the optimal value, and finally, 10 risk factors including gender, household registration, education, pain, drinking, life satisfaction, self-rated memory, self-rated health, number of chronic diseases and age were screened out.

### Construction of LR risk prediction model

**Figure 4:**
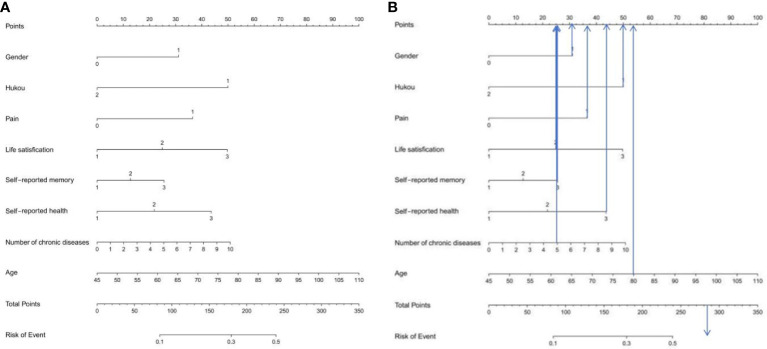
**(A)** Nomogram prediction model of logistic risk of depression in middle-aged and elderly patients with hypertension **(B)** Score explanation of the incidence of depression in middle-aged and elderly patients with hypertension. According to the risk factors in the Nomogram, the corresponding score of each variable can be known. After adding the scores of each variable, the prediction probability corresponding to the total score is the probability of depression in middle-aged and elderly hypertensive patients. Points is the individual score, Total Points is the total score, and Risk of Event is the incidence of depression corresponding to the total score.

After selection by lasso regression, we performed a multivariate logistic regression analysis in the training set, using forward stepwise and likelihood ratio tests to rule out confounding factors. The results are shown in **Table 2**, gender, hukou, pain, life satisfaction, self-reported health, self-reported memory, number of chronic diseases and age are independent risk factors for depression in middle-aged and elderly patients with hypertension. We plotted the nomogram for depression in middle-aged and elderly hypertensive patients as shown in **Figure 4A**, and the specific variable allocation is shown in **Table 3**. Finally, based on the risk factor scores in the nomogram, we can calculate the corresponding predicted probability, which is the probability of depression in middle-aged and elderly hypertensive patients. For example, female (score 31), agricultural hukou (score 50), pain (score 36.5), fair life satisfaction (score 25), poor self-rated memory (score 20.5), poor self-rated health (score 43), 5 chronic diseases (score 25), and age 80(score 54). These scores are added to give a total score of 285, corresponding to a risk of more than 50%. This means that patients may be at higher risk for depression and therefore require individualized preventive measures. The specific scoring criteria are shown in **Figure 4B**.

**Table 2:** Multivariate logistic analysis of depression in middle-aged and elderly patients with hypertension.

Variables	Coef	S.E.	Wald *Z*	*p*
**Gender**	0.439	0.108	4.08	<0.001
**Hukou**	-0.706	0.129	-5.47	<0.001
**Pain**	0.516	0.117	4.42	<0.001
**Life satisfication**	0.351	0.095	3.68	<0.001
**Self-reported health**	0.308	0.090	3.41	<0.001
**Self-reported memory**	0.181	0.089	2.03	0.043
**Number of chronic diseases**	0.072	0.033	2.16	0.031
**Age**	0.022	0.006	3.58	<0.001

**Table 3:** Case of variable assignment.

Variable	Assignment mode
**Gender**	Male=0; Female=1
**Hukou**	Agricultural hukou=1; Non-agricultural hukou=2
**Pain**	No=0; Yes=1
**Life satisfication**	Good=1; Fair=2; Bad=3
**Self-reported health**	Good=1; Fair=2; Bad=3
**Self-reported memory**	Good=1; Fair=2; Bad=3
**Number of chronic diseases**	Measured value
**Age**	Measured value

### Construction of RF risk prediction model

**Figure 5:**
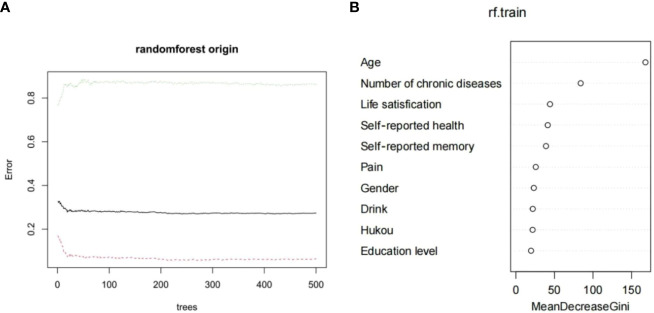
**(A)** Dynamic relationship between prediction error of random forest and the number of random trees **(B)** Importance ranking of variable factors based on random forest.

A total of 2050 samples were used to establish the random forest model in the training group. Based on the value of mtry is 5, when ntree=500, the error basically tends to be stable, and the dynamic relationship between the prediction error of random forest and the number of random trees is shown in **Figure 5A**. Therefore, it is the optimal model when mtry=6 and mtree=500. According to the mean value of Gini index reduction, the importance of the 10 variables was ranked. Age, number of chronic diseases, life satisfaction, self-rated health, and self-rated memory were the top 5 important indicators for predicting depression in middle-aged and elderly hypertensive people, as shown in **Figure 5B**.

### Construction of XGBoost risk prediction model

**Figure 6:**
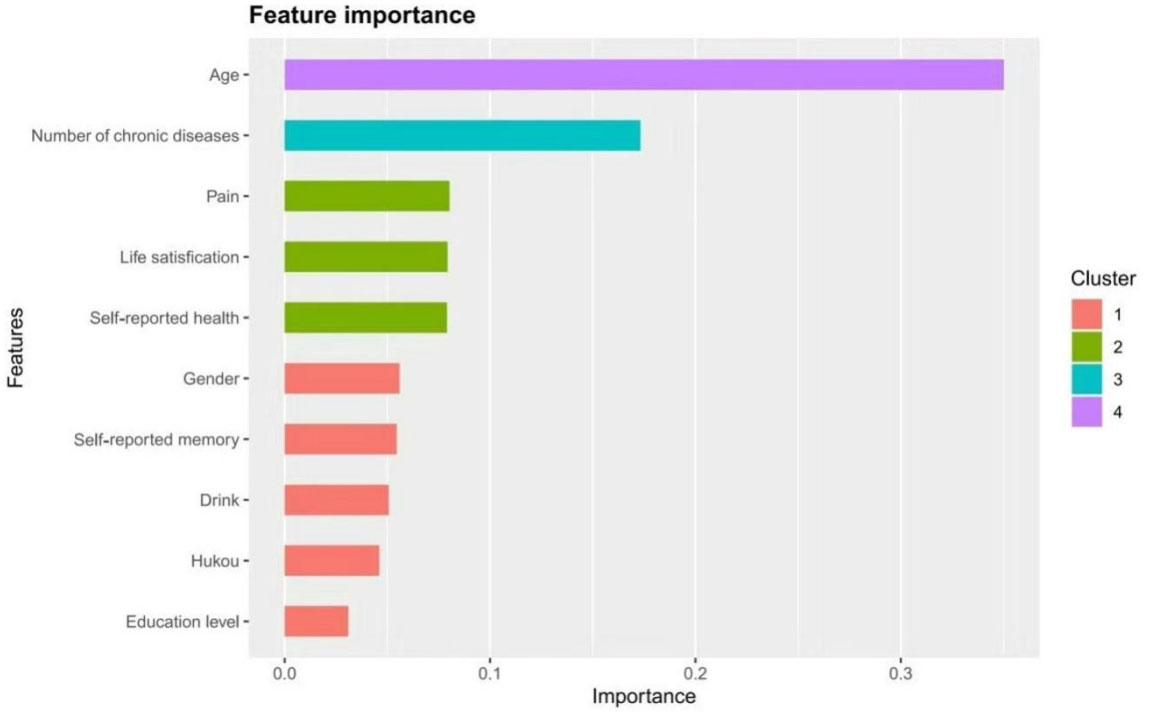
Importance ranking of variable factors based on XGBoost.

In this study, risk factors were input into the XGBoost model for analysis. From the feature importance results in **Figure 6**, it can be seen that the top eight influencing factors are age, number of chronic diseases, pain, life satisfaction, self-rated health, gender, self-rated memory, and drink. Based on these results, the first eight factors were selected as input features for the model in this study.

### Performance comparison results of three prediction models

**Figure 7:**
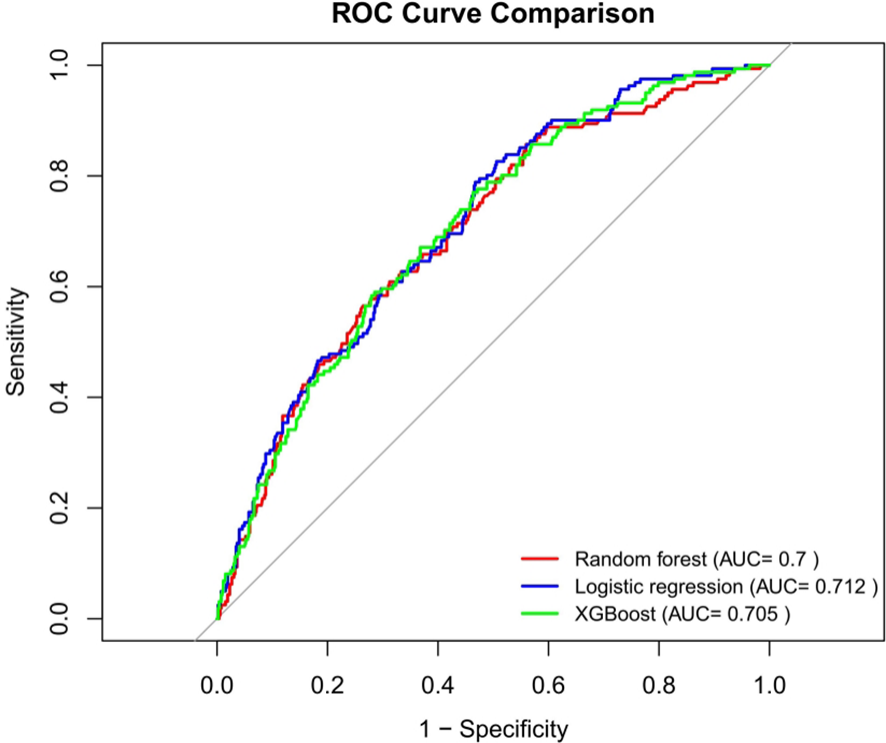
ROC curves for the three models.

In this study, we used the 683 participants in the testing set to compare the predictive effects of RF, LR and XGBoost three models on depression in middle-aged and elderly hypertensive people. As shown in **Figure 7**, the AUCs of the three models in the testing set ranged from 0.7 to 0.712. The AUC value of LR was the highest (AUC=0.712), and the AUC value of RF was the lowest (AUC=0.7). We also evaluated the accuracy, sensitivity, specificity, and other performance indicators of the model, as detailed in **Table 4**. The thresholds of the three models were 0.271, 0.191 and 0.198, respectively. The sensitivity of the LR model was the highest (0.789), followed by the XGBoost model (0.770), and the NPV of the LR model was the highest (0.716). In general, the LR model performed the best.

**Table 4:** Comparison of prediction performance of two kinds of models.

Model	AUC	Sensitivity	Specificity	PPV	NPV
**Random forest**	0.7	0.565	0.736	0.681	0.629
**Logistic regression**	0.712	0.789	0.533	0.628	0.716
**Xgboost**	0.705	0.770	0.536	0.624	0.700

## Discussion

### Comparative model analysis

According to the 10 selected variables, this study used LR, RF and XGBoost machine learning algorithms to construct the risk prediction models for depression in middle-aged and elderly hypertensive patients and compared the models. The results showed that the LR-based model had the best performance in the area under the ROC curve, sensitivity and NPV, and was the best in general. In contrast, RF had the worst combined performance in several models. Several other machine learning algorithms are better able to deal with complex nonlinear relationships between variables, and using multiple algorithms can find the algorithm that performs best in this dataset, which is helpful to find models with good performance. In addition, we also constructed a nomogram model, which can be used to calculate the risk value of depression in middle-aged and elderly hypertensive patients. For example, female (score 31), agricultural hukou (score 50), pain (score 36.5), fair life satisfaction (score 25), poor self-rated memory (score 20.5), poor self-rated health (score 43), 5 chronic diseases (score 25), and age 80(score 54). These scores are added to give a total score of 285, corresponding to a risk of more than 50%. This means that patients may be at higher risk for depression and therefore require individualized preventive measures.

### Influencing factors of depression in middle-aged and elderly patients with hypertension

This study found that depression in middle-aged and elderly patients with hypertension increased with age. This is consistent with the results of previous studies (43–46) and may be caused by factors such as the decline of physiological function and the weakening of psychological tolerance. Maatouk et al. interviewed 3,124 participants aged 57 to 84 years and used Logistic regression analysis to explore the relationship between high blood pressure and depressive symptoms (17). The findings show a significant link between depression and high blood pressure. In addition, individual perception is also influenced by many factors such as society, family, surrounding environment, economic income, culture, and customs (47). This suggests that we need to pay more attention to the mental health of middle-aged and elderly people, especially patients with hypertension. Therefore, in future work, more attention should be paid to the mental health of middle-aged and elderly people with hypertension to alleviate their negative emotions such as depression and anxiety. In addition, female patients were more likely to have depressive symptoms than men, which is consistent with the results of most studies (29, 48, 49). This may be due to the fact that women have a lower psychological tolerance to cope with stress, and are more sensitive to negative emotions, and a higher neuroticism score is also a factor (50). In addition, women often bear dual responsibilities and stress at home and at work, and women with chronic diseases may face difficulties in fulfilling gender-specific social roles, which also increases their risk of depressive and anxiety symptoms (48).

There is also a large difference in depressive symptoms between middle-aged and elderly hypertensive patients in urban and rural areas. Middle-aged and elderly hypertensive patients with agricultural hukou are more likely to have depressive symptoms, which is similar to the results of related studies (51). According to this study, nearly half (40.6%) of the elderly hypertensive patients in rural areas of Jinzhong City of Shanxi Province, China were assessed using the CES-D (Center for Epidemiologic Studies Depression Scale), and nearly half (40.6%) of the patients had depressive symptoms (52). The reasons for this difference may be multifactorial. First of all, the economic level of middle-aged and elderly people in rural areas is much lower than that in towns and cities, which leads to the imperfect living environment, medical level and social security. Due to the limitation of economic conditions, the decrease of life status and satisfaction may increase the incidence of depression in middle-aged and elderly people in rural areas (53). Secondly, hypertensive patients in rural areas have lower awareness of disease, distance from medical institutions, and access to medical resources. This results in a lower sense of well-being for the patient, which in turn triggers depressive feelings.

Therefore, in addition to considering the disease factors of patients themselves, the government can start from the supply of public services and diagnosis and treatment services to formulate relevant policies (54). At the same time, according to the differences between urban and rural areas, different strategies can be taken to improve the mental health status of middle-aged and elderly patients with hypertension in rural areas. This may include better medical resources, better awareness of the disease, and a better social safety net. Through these measures, better support and help can be provided to middle-aged and elderly patients with hypertension and their quality of life can be improved (55).

Patients with hypertension often have other chronic diseases, and the results of this study suggest that those with coexisting chronic diseases are more likely to have depressive symptoms, and the more chronic diseases they have, the more likely they are to have depressive symptoms. This is consistent with other findings (48, 56) and may be due to limitations in daily functioning related to coexisting conditions (48). A similar conclusion was reached in the study by Gray et al. (57), who found that diabetes was an independent predictor of concurrent hypertension and depressive symptoms. In addition, cerebrovascular complications such as stroke and intracerebral hemorrhage were also more common in the depressed group, and many studies have shown that the occurrence of stroke is associated with an increased risk of depressive symptoms (58). Therefore, more attention should be paid to the emotional state of middle-aged and elderly hypertensive patients with multiple diseases in clinical practice.

The results of this study also showed that depressive symptoms were further aggravated if hypertensive patients also suffered from pain. This is similar to the findings of Chen et al (59) and Gerrits et al (60). In addition, a large number of cross-sectional studies have found that patients with pain have an increased risk of depression (61), and similarly, patients with depression have a higher risk of pain than those without depression (62). This may be because pain causes people to be limited in their physical activities, affecting their lives and reducing the frequency of contact with others. As pain and lack of social activities increase psychological distress, this leads to an increased incidence of depression. In conclusion, middle-aged and elderly patients with hypertension often have other chronic diseases, and the coexistence of these chronic diseases as well as pain symptoms may increase the risk of depression. Therefore, these complications should be paid attention to and actively managed in clinical practice to improve the mental health of patients.

Life satisfaction and depressive symptoms are two psychological constructs that are widely recognized and are often used in population health surveys to assess mental health (63). Europe and the United Kingdom and other countries have used life satisfaction as a relevant indicator to evaluate mental health (64). Although relatively few studies have examined the association between life satisfaction and blood pressure, the association has been found to be somewhat protective (65–68). The present study found that middle-aged and elderly hypertensive patients are more likely to have depressive symptoms, which is consistent with the results of previous studies (69). This may be due to the fact that middle-aged and elderly patients with low life satisfaction may experience many negative experiences and life stresses, which can exacerbate depressive moods. Therefore, when helping older people cope with depression, we need to focus on their life satisfaction and quality of life, provide positive support and help to help them cope with negative emotions and stress, and improve their quality of life and mental health.

The results of the present study also showed that patients with poorer self-rated memory had more severe depressive symptoms. Many previous studies have shown that people with high blood pressure have a decline in memory (70, 71). In addition, the results of a 12-year follow-up showed that middle-aged hypertensive people had worse cognitive function than healthy people without hypertension during the same period, and untreated hypertensive people had worse memory and information processing speed than those with stable blood pressure control (71). Subsequently, depressive symptoms occur due to memory loss in hypertensive patients, which has been demonstrated in previous studies (72, 73). Therefore, cognitive function training can be carried out in advance for middle-aged and elderly patients with hypertension in the future, which will contribute to the mental health of patients. In addition, poorer self-rated health was associated with a higher risk of depression. This is similar to the findings of Boima et al (43). On the one hand, it may be because when the self-rated health is poor, the ability of daily life is affected and the quality of life is decreased, resulting in heavier psychological burden, greater pressure, and more likely to produce depressive symptoms. On the other hand, it may be because the decline of health may lead to the limitation of people’s communication and social activities with others. Lack of social support and a sense of isolation, which can also lead to the onset and exacerbation of depressive mood.

## Limitations

There are some limitations in this study. First, we were unable to include all possible risk factors due to the structural limitations of the questionnaire. Second, the data relied heavily on respondents’ self-reports, which may be subject to memory bias, subjective interpretation, and social expectations. Finally, although several methods were used to ensure the reliability and generalizability of the model, the results still need to be validated in external independent samples. Despite these limitations, we believe that these findings are instructive for the prevention and intervention of depressive symptoms in middle-aged and elderly patients with hypertension. Future research could further collect data on related factors to make the analysis of factors affecting depression more comprehensive and in-depth.

## Conclusion

In this study, a variety of machine learning algorithms were used to construct models for predicting the risk of depression in middle-aged and elderly patients with hypertension. After evaluation and comparison, the LR model was found to be the most predictive model. The results showed that gender, hukou, pain, life satisfaction, self-reported health, self-reported memory, number of chronic diseases, and age are risk factors for depression in middle-aged and elderly hypertensive patients. Medical staff can formulate intervention programs based on these related risk factors and implement them as early as possible to reduce the adverse effects of depression on middle-aged and elderly patients with hypertension.

## Data availability statement

Publicly available datasets were analyzed in this study. This data can be found here: The datasets presented in this study can be found in online repositories. The names of the repository/repositories and accession number(s) can be found below: The datasets generated for this study can be found in the China Health and Retirement Longitudinal Study (CHARLS) online datasets (https://charls.charlsdata.com/pages/data/111/zh-cn.html).

## Ethics statement

The studies involving humans were approved by the Institutional Review Board of Peking University (IRB00001052-11015). The studies were conducted in accordance with the local legislation and institutional requirements. The participants provided their written informed consent to participate in this study.

## Author contributions

FA: Conceptualization, Data curation, Writing – original draft, Writing – review & editing. EL: Conceptualization, Writing – original draft, Writing – review & editing. HZ: Data curation, Writing – review & editing. QJ: Data curation, Writing – original draft.
